# Editorial: Hepatocellular carcinoma: from personalized medicine to practical guidelines

**DOI:** 10.3389/fphar.2023.1301202

**Published:** 2023-11-02

**Authors:** Sarah El-Nakeep, Anup Kasi

**Affiliations:** ^1^ Hepatogastroenterology Unit, Internal Medicine Department, Faculty of Medicine, Ain Shams University, Cairo, Egypt; ^2^ Cancer Center, University of Kansas, Kansas City, MO, United States

**Keywords:** HCC, personalized medicine, molecular pathway, biomarkers, current guidelines

## Introduction

Hepatocellular carcinoma (HCC) is the most common primary liver malignancy, with a survival rate of 18%. The recent Barcelona 2022 guidelines updated the liver transplantation category, including a downstaging group by transarterial chemoembolization (TACE) and *first-line* combination therapy with atezolizumab and bevacizumab for advanced HCC (aHCC) (Tsilimigras et al., 2022). These small steps towards increasing the options for patients who are considered advanced pave the way for improving survival. Molecular and immunotherapeutic drugs are available only for aHCC (Reig et al., 2022) with low survival benefits (Sun H. et al.). Moreover, there is no preferred regimen for the first- or second-line treatment of aHCC, despite multiple approved Food and Drug Administration (FDA) molecules. A policy review suggested a multiparametric therapeutic hierarchy, from a surgical approach to systemic therapy, offered according to the survival benefit to the individual patient, was proposed recently (Vitale et al., 2023). Personalized medicine using specific molecular pathway modifications is limited to vascular endothelial growth factor inhibitors (VEGFI), tyrosine kinase inhibitors (TKI), and immune checkpoint inhibitors (ICI). A meta-analysis using ICI combined with VEGFI as first-line therapy for aHCC, on 3168 patients, showed that this combination was safe and tolerable, with a pooled median OS of 14.7 months (Gao et al., 2023). The combination of local and systemic approaches is promising. A cohort showed a median OS of 21.8 months with the combination of sorafenib and local hepatic artery infusion of chemotherapy after TACE (Liu et al., 2020).

**FIGURE 1:**
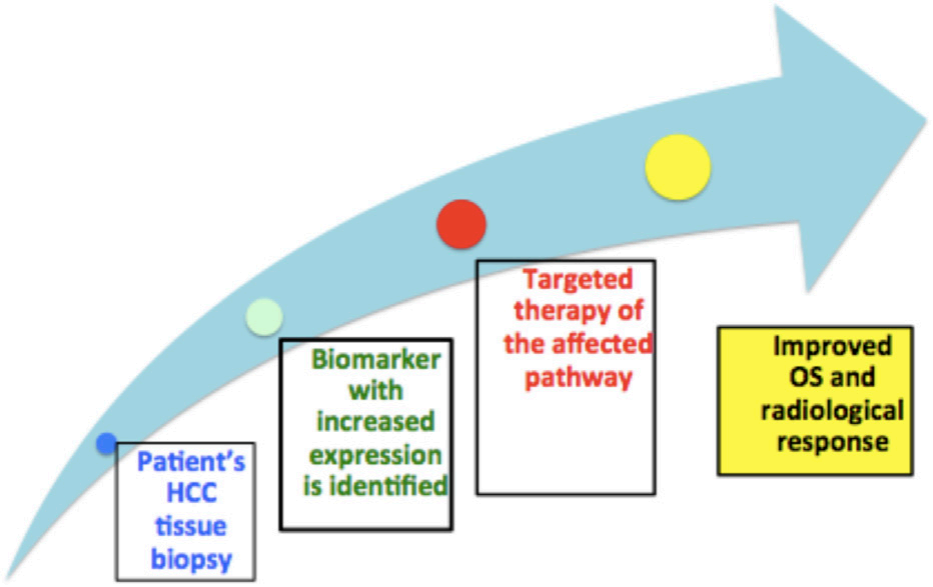
Proposed personalized approach and points of limitations.

Limitations of personalized medicine are illustrated in Figure 1. First, prior availability of histopathological studies is lacking, as tissue biopsy is not a common procedure in HCC cases, only retrospective specimens are available from resection.

Second, biomarker expression is mostly heterogeneous, with multiple affected molecular pathways and genetic mutations and no biomarker-driven therapy pathway, such as in lung and colon cancers, is determined. Moreover, patients with low or negatively expressed biomarkers may respond to targeted therapy similar to those with positively expressed biomarkers (Chan et al., 2022).

Additionally, cirrhosis surrounding the cancer with different sets of mutated molecular pathways caused by chronic inflammation needs to be treated differently from cancer (Chan et al., 2022).

Third, resistance to targeted therapy occurs owing to intratumoral heterogeneity, as each area has different molecular pathway mutation; resulting in tumor “flare,” and worsening the prognosis (Chan et al., 2022).

Finally, “financial toxicity” (Gyawali, 2017) is of concern given the ethical issue of comparing new drugs to placebo, and not the standard treatment, or the clinical preference of drugs with a meager increase in survival by 1–3 months over that of the standard therapy, with a higher cost.

Hope lies in personalized medicine. First, is the ability to decide beforehand which molecular pathway modification is most suitable according to genetic or molecular testing. Only one FDA-approved test using next-generation sequencing for tumor profiling includes 468 genes (MSA-IMPACT) (Jibiki et al., 2021). The test detects drug resistance using sorafenib and immunotherapy (Dominguez and Wang, 2020). Only one biomarker-targeted therapy, ramucirumab, is FDA-approved after the REACH-2 trial, which increases the OS for patients with aHCC who failed sorafenib with an AFP ≥ 400 ng/mL. The placebo group had an OS of 7.3 months vs. 8.5 months in the active group (Zhu et al., 2019). However, its mechanism of action remains unclear.

Second, drug docking was used to identify novel molecular pathway drugs. Artificial intelligence could be crucial in future testing of molecular models, decreasing the time required for preclinical validation.

Our Research Topic includes five reviews, two cohort studies, a bibliometric and case study. Guan et al. present an excellent review which discusses second-line switching in aHCC using TKI, VEGF, and/or ICIs after failure of the first combination treatment. Single switching is shown to be better than double drug switching in terms of OS and disease progression. Moreover, lenvatinib retention improved survival after switching to a single drug.

The cohort study by Lei et al. comparing TKI monotherapy to TKI combined with PD-1 as a secondary treatment after sorafenib failure, resulted in improved median OS of 21.9 months in the combination group vs. 16.6 months in the monotherapy group.

Another continuous cohort study by Li et al. explored the use of lenvatinib in post-viral HCC; a higher efficacy was observed on HBV-infected than on HCV-infected patients.

An informative review article by Sun L. et al. discusses immune-related adverse events (irAEs) of various immunotherapies. The most common irAEs were cutaneous, gastrointestinal, and hepatic. PD-1 and PD-L1 inhibitors caused dose-independent irAEs, whereas CTLA-4 inhibitors caused dose-dependent irAEs. This review highlights the need for conducting further research on the importance of irAEs as a limiting factor in the treatment of patients, resulting in withdrawal or decrease in the dose of the drug.

The valuable bibliometric study by Wang et al. provides a scientometric analysis of the research published on lenvatinib in HCC and shows an annual growth of 102.5% in this Research Topic.

The review by Sun H. et al. is of practical use and discusses the available targeted therapies in medical practice. First- and second-line therapies approved by the FDA, including TKI, VEGFI, and PD-1 inhibitors used as monotherapy provide a 14–16 months survival rate with serious dose-related side effects and drug resistance. Combination therapy between different categories showed a safety profile similar to that of monotherapy, with some improvement in survival rates by 4–6 months. However, the optimal combination regimen remains undetermined. Bioengineering in the form of patient-derived organoids, patient-derived xenografts, and 3D printing could allow for further personalized approaches.

The detailed review article by Xiao et al. focuses on the role of hypoxia-inducible factors in the recurrence of HCC after ablation by inducing the VEGF pathway.

The minireview by Jiang et al. discusses immunotherapy in post-liver transplantation recurrence. The authors recommended caution in using immunotherapy in this category, as it may result in graft rejection.

Finally, Park et al. present the promising case of a 57-year-old male patient with aHCC treated with a combination of immunotherapy and the anticancer herbal extract Gun-Chil-Jung, who had an OS of 20.3 months.

Overall, our Research Topic covers a snippet of advances in personalized medicine in the literature and highlights the need for further drug research, weighing the balance between survival benefits and hazardous adverse events.

## References

[B1] Chan S. L. Wong N. Lam W. K. J. Kuang M. (2022). Personalized treatment for hepatocellular carcinoma: current status and future perspectives. J. Gastroenterol. Hepatol. 37 (7), 1197–1206. 10.1111/jgh.15889 35570200

[B2] Dominguez D. A. Wang X. W. (2020). Impact of next-generation sequencing on outcomes in hepatocellular carcinoma: how precise are we really? J. Hepatocell. Carcinoma 7, 33–37. 10.2147/jhc.s217948 32257970 PMC7090189

[B3] Gao X. Zhao R. Ma H. Zuo S. (2023). Efficacy and safety of atezolizumab plus bevacizumab treatment for advanced hepatocellular carcinoma in the real world: a single-arm meta-analysis. BMC Cancer 23 (1), 635. 10.1186/s12885-023-11112-w 37415136 PMC10327339

[B4] Gyawali B. (2017). Low-value practices in oncology contributing to financial toxicity. Ecancermedicalscience 11, 727. 10.3332/ecancer.2017.727 28386297 PMC5365336

[B5] Jibiki T. Nishimura H. Sengoku S. Kodama K. (2021). Regulations, open data and healthcare innovation: a case of MSK-IMPACT and its implications for better cancer care. Cancers (Basel) 13 (14), 3448. 10.3390/cancers13143448 34298662 PMC8304506

[B6] Liu B. J. Gao S. Zhu X. Guo J. H. Zhang X. Chen H. (2020). Sorafenib combined with embolization plus hepatic arterial infusion chemotherapy for inoperable hepatocellular carcinoma. World J. Gastrointest. Oncol. 12 (6), 663–676. 10.4251/wjgo.v12.i6.663 32699581 PMC7341000

[B7] Reig M. Forner A. Rimola J. Ferrer-Fàbrega J. Burrel M. Garcia-Criado Á. (2022). BCLC strategy for prognosis prediction and treatment recommendation: the 2022 update. J. Hepatology 76 (3), 681–693. 10.1016/j.jhep.2021.11.018 PMC8866082 34801630

[B8] Tsilimigras D. I. Aziz H. Pawlik T. M. (2022). Critical analysis of the updated Barcelona clinic liver cancer (BCLC) group guidelines. Ann. Surg. Oncol. 29 (12), 7231–7234. 10.1245/s10434-022-12242-4 35854026

[B9] Vitale A. Cabibbo G. Iavarone M. Viganò L. Pinato D. J. Ponziani F. R. (2023). Personalised management of patients with hepatocellular carcinoma: a multiparametric therapeutic hierarchy concept. Lancet Oncol. 24 (7), e312–e322. 10.1016/s1470-2045(23)00186-9 37414020

[B10] Zhu A. X. Kang Y. K. Yen C. J. Finn R. S. Galle P. R. Llovet J. M. (2019). Ramucirumab after sorafenib in patients with advanced hepatocellular carcinoma and increased α-fetoprotein concentrations (REACH-2): a randomised, double-blind, placebo-controlled, phase 3 trial. Lancet Oncol. 20 (2), 282–296. 10.1016/S1470-2045(18)30937-9 30665869

